# Acromioclavicular joint reconstruction using a tendon graft: a biomechanical study comparing a novel “sutured throughout” tendon graft to a standard tendon graft

**DOI:** 10.1051/sicotj/2016013

**Published:** 2016-04-20

**Authors:** Qais Naziri, Nadine Williams, Westley Hayes, Bhaveen H. Kapadia, Dipal Chatterjee, William P. Urban

**Affiliations:** 1 Department of Orthopaedics, SUNY Downstate Medical Center 450 Clarkson Avenue, MSC 30 Brooklyn NY 11203 USA

**Keywords:** Biomechanics, Acromioclavicular joint, UHMWPE suture, Coracoclavicular ligament, Graft augmentation

## Abstract

*Background*: With a recurrence rate of over 30%, techniques that offer stronger acromioclavicular (AC) joint reconstruction through increased graft strength may provide longevity. The purpose of our study was to determine the biomechanical strength of a novel tendon graft sutured throughout compared to a native tendon graft in Grade 3 anatomical AC joint reconstruction.

*Methods*: For this in vitro experiment, nine paired (*n* = 18) embalmed cadaveric AC joints of three males and six females (age 86 years, range 51–94 years) were harvested. Anatomic repair with fresh bovine Achilles tendon grafts without bone block was simulated. Specimens were divided into two groups; with group 1 using grafts with ultra-high molecular-weight polyethylene (UHMWPE) suture ran throughout the entire length. In group 2, reconstruction with only native allografts was performed. The distal scapula and humerus were casted in epoxy compound and mounted on the mechanical testing machine. Tensile tests were performed using a mechanical testing machine at the rate of 50 mm/min. Maximum load and displacement to failure were collected.

*Results*: The average load to failure was significantly higher for group 1 compared to group 2, with mean values of 437.5 N ± 160.7 N and 94.4 N ± 43.6 N, (*p* = 0.001). The average displacement to failure was not significantly different, with 29.7 mm ± 10.6 mm in group 1 and 25 mm ± 9.1 mm in group 2 (*p* = 0.25).

*Conclusion*: We conclude that a UHMWPE suture reinforced graft can provide a 3.6 times stronger AC joint reconstruction compared to a native graft.

## Introduction

Acromioclavicular (AC) joint injuries are common in the active population [1–3]. The injury often involves direct trauma to the superior aspect of the acromion, which causes an inferior and anterior translation of the acromion in relation to the distal clavicle [4]. Conservative management is used for the treatment of Rockwood Type I–III (in nonathletes) separations [3, 5]. Operative management is often indicated in athletes and for chronic symptomatic Type III separations that have not responded to conservative treatment. Type IV–VI AC separations generally require operative management [1].

More than 60 surgical procedures to treat AC joint separation have been reported. These can be categorized into four different types which are (1) primary AC and coracoclavicular (CC) fixation, (2) the Weaver-Dunn procedure, (3) anatomic reconstruction, and (4) arthroscopic reconstructions [6].

Primary AC and CC fixation accomplished with Kirschner wires, sutures, and Bosworth screws are one of the first fixation methods. Though good results have been reported, these methods have fallen into disfavor due to hardware migration, of which some have been devastating [7–9]. The proper Weaver-Dunn procedure demands the excision of the distal clavicle and transfer of the CA ligament onto the CC ligament. Biomechanically this construct seemed weaker and led to greater displacement [10].

Subsequently, this method has been more commonly augmented with sutures, wire cerclage, or tendon grafts for reinforcement. In order to replicate biomechanical behavior of the native ligament, anatomic reconstructions, wherein the conoid and trapezoid ligaments are recreated, have become more popular [2]. However, anatomic reconstruction has been reported to fail via coracoid process fractures, clavicular fractures of the bone tunnels, or AC joint separations [10, 11]. Newer arthroscopic techniques involve suture and screw fixation. In a comparative study by Mazzocca et al. arthroscopic fixation showed less anterior displacement and laxity than the Weaver-Dunn procedure, compared to anatomic reconstructions the outcomes were similar [12]. Open procedures are becoming increasingly harder to justify due to the greater risk of iatrogenic injury to adjacent neurovascular structures [13].

So far, the orthopedic literature does not contain recommendations for a single operative technique as the optimal reconstruction method for AC joint separations. Postsurgical recurrence rates of AC joint separation after reconstruction can range between 20 and 30% or higher and occur frequently within one year of the initial surgery [14–18]. Recently the focus has been on reconstruction techniques utilizing tendon grafts, as the need for implant removal is obviated and implant fracture, loosening, and migration are eliminated. Therefore, various tendon grafts, including palmaris longus, semitendinosus, anterior tibialis, or gracilis tendon, have been utilized [4, 19, 20]. However, one of the limiting factors for the longevity of reconstruction techniques using tendon grafts is the biomechanical strength of the tendon graft itself as they do not reestablish the strength and stiffness of the native AC joint [21–23]. Several authors have advocated more resilient fixation to improve surgical outcomes [11, 24–26].

While tendon graft repair techniques augmented with cerclage cables have been described, to our knowledge, no study has evaluated the effect of an intra-tendinous suture that functions similarly to a coracoid cerclage construction, on the strength of the AC joint repair [27].

We hypothesized that running a suture throughout the tendon graft itself would increase load to failure and the longevity of reconstruction techniques using tendon grafts. Therefore, the purpose of our study was to determine the difference in tensile strength and displacement to failure, between a novel tendon graft with ultra-high molecular-weight polyethylene (UHMWPE) suture incorporated throughout and a standard nonaugmented tendon graft in an anatomic AC joint reconstruction.

## Material and methods

For this biomechanical laboratory study evaluating tensile load to failure and displacement to failure of a novel graft augmentation technique, nine paired (*n* = 18) cadaveric AC joints (three males and six females) were harvested. After thorough inspection, none of the 18 specimens showed signs of prior injury to the shoulder or prior operative intervention and thus were all included for biomechanical testing. The mean age of the embalmed cadaveric donors at the time of death was 86 years (range, 51–94 years). Surrounding muscles and other soft tissues were removed. The AC and coracoclavicular (CC) ligaments were severed in order to simulate a Type III dislocation.

## Specimen preparation

**Figure 1.**
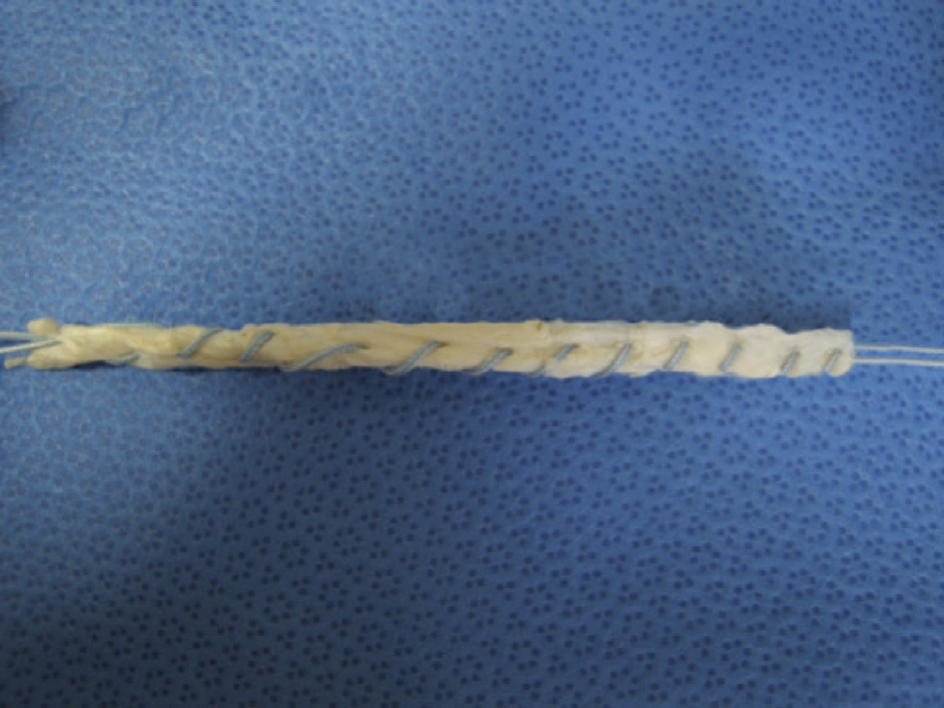
Graft with UHMWPE suture ran throughout the entire length for reinforcement.

Fresh-frozen bovine Achilles tendon grafts, which measured in average 5 mm × 127 mm, were obtained. All tendon specimens were stored at −20 °C for no longer than four weeks and thawed at room temperature for 12 h prior to testing. Nine grafts were whipstitched with UHMWPE suture (#5 FibreWire^TM^, Arthrex, Naples, FL) across the entire length (Figure 1) on a graft preparation station. All specimens were wrapped in saline-soaked gauze.

The clavicular bone tunnel sites were carefully and accurately measured prior to drilling as previously described by Carofino and Mazzocca [2].

A drill tip guide pin was used for placement of the tunnels. The first tunnel was created in the posterior aspect of the clavicle approximately 45 mm from the distal end. After measuring 20 mm distally from the center of the first tunnel, the second tunnel was created in the anterior aspect of the clavicle. Both tunnels were then reamed through the full thickness of the clavicle using a 5.5 mm reamer.

The matched pairs of AC joints were separated. Randomly, either the left or the right shoulder was assigned a graft sutured throughout with UHMWPE suture and became group 1 (*n* = 9). The contralateral AC joint received the standard native bovine Achilles tendon graft and represented group 2 (*n* = 9). Prior to joint reconstruction all grafts were tensioned to 10 N to remove any creep.

**Figure 2.**
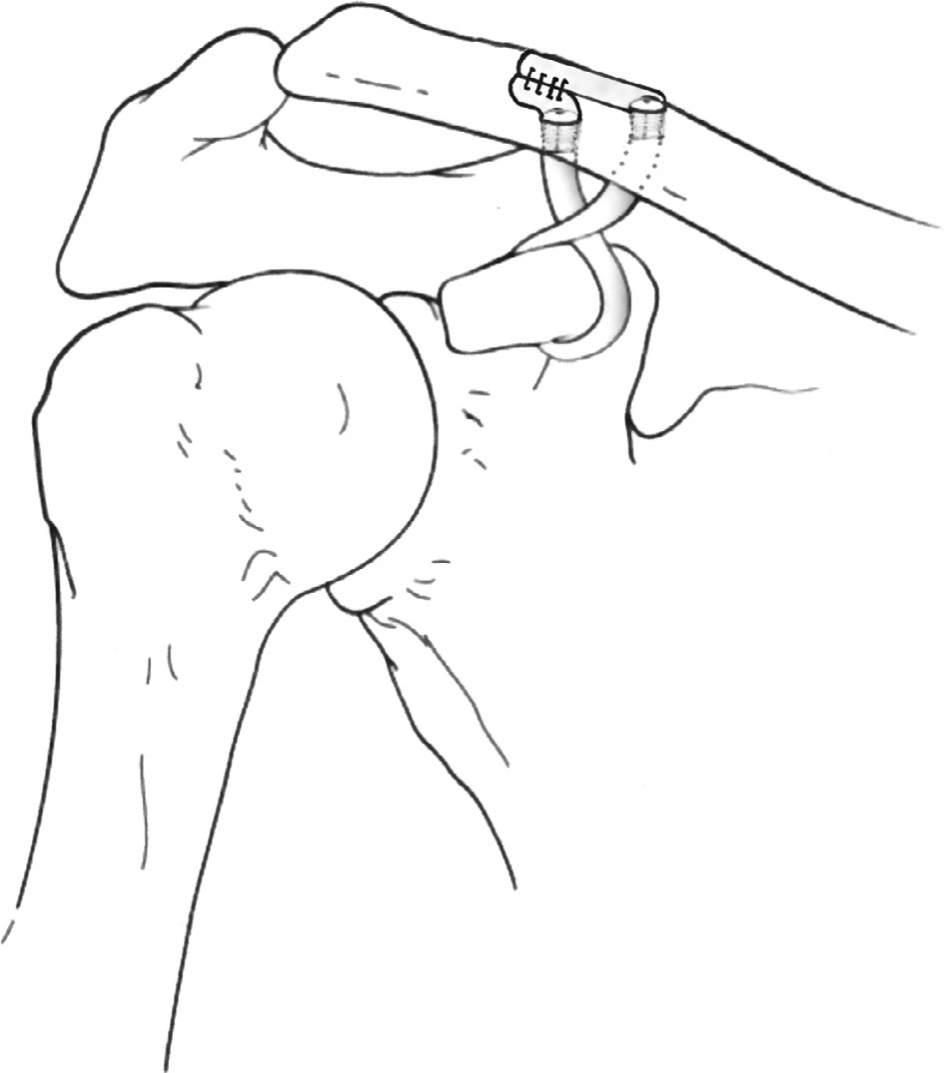
Schematic diagram of anatomic AC joint reconstruction. In group 1 suture was placed into the entire graft (not depicted) while group 2 lacked suture within the graft.

In both groups, the tendon graft was looped around the base of the coracoid process. Then the lateral limb of the tendon graft was placed through the posteromedial bone tunnel, which recreated the conoid ligament medially. The medial limb of the graft was passed through the anterolateral bone tunnel, which recreated the trapezoid ligament laterally. This method of passing the graft through creates a crossing pattern as described by Shin et al. [27]. In all specimens the grafts were fixed in the bone tunnels using 5.5 mm × 8 mm Bio-Tenodesis Screws (PEEK, Arthrex, Naples, FL). The ends of the graft were sutured to each other in a side-to-side fashion in both groups (Figure 2).

**Figure 3.**
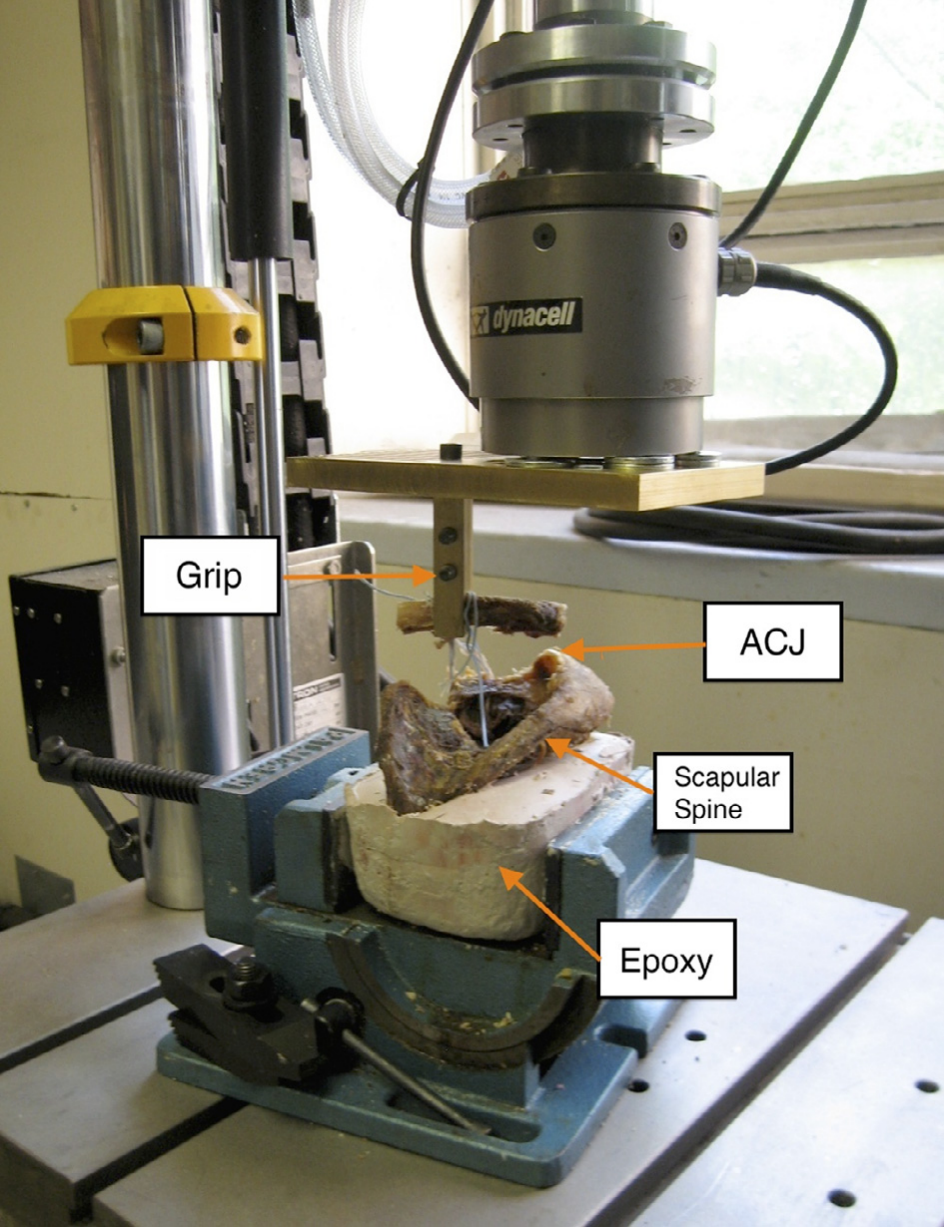
The testing construct, which is attached to the load cell and the base of the mechanical testing machine.

The distal ends of the scapula and humerus were casted in epoxy compound (Bondo^TM^, Atlanta, GA). The epoxy end was mounted in a shop vise that was bolted to the mechanical testing machine (Instron^TM^ 8874 Biaxial Testing Machine, Norwood, MA) with calibrated load cells. Custom-made fixture and grips, made from a bronze alloy ASTM B150, were used to grab the clavicle (Figure 3). The *Z*-axis was defined as the tensile axis and was arranged longitudinally to the tendon graft. Tensile test was performed at a deformation rate of 50 mm/min. Maximum load to failure and displacement to failure were collected. Similar to Elenes et al. failure was defined at the breaking point of the failure test curve [28]. Samples that experienced fracture of the clavicle during loading were excluded from statistical analysis.

Data was stored in Microsoft Excel Spreadsheet (Microsoft Corp, Redmond, Washington). For statistical analysis a paired Student’s t test was performed using SPSS 22.0 (2013, IBM Corp, Armonk, NY).

## Results

**Figure 4.**
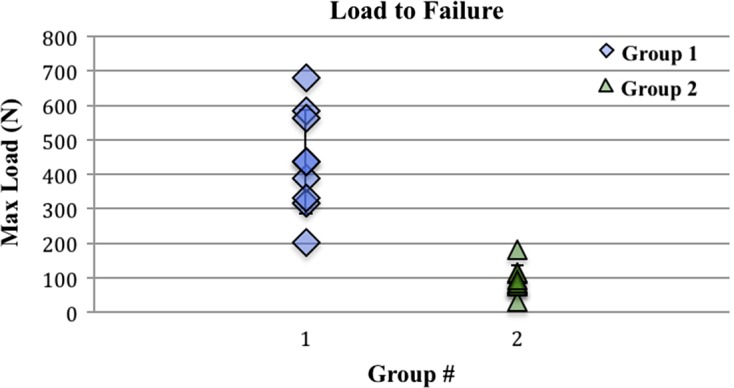
Maximum load to failure of eight specimens in each group. Difference between groups is statistically significant (*p* = 0.001).

**Figure 5.**
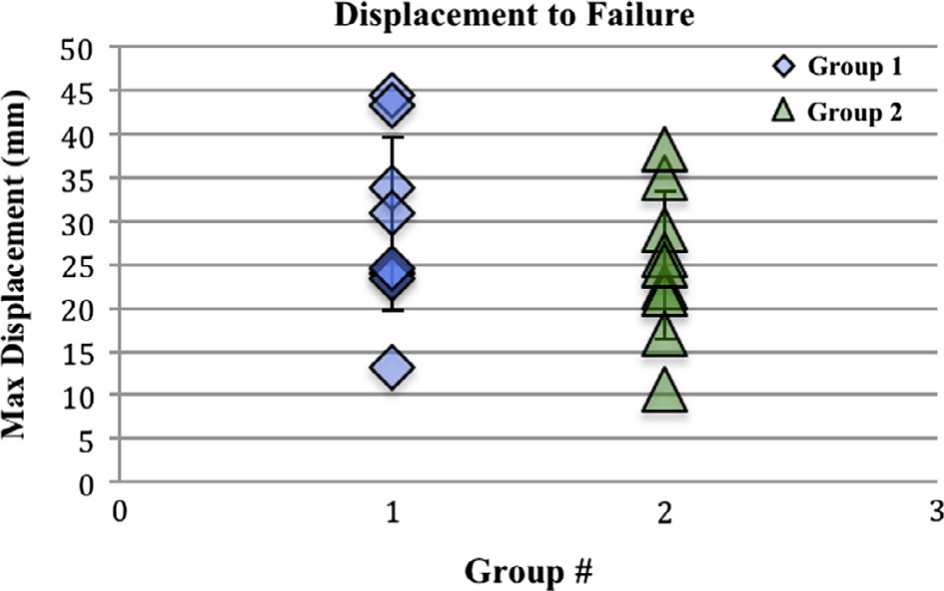
Displacement to failure of eight specimens in each group. Difference between groups is not statistically significant (*p* = 0.25).

All failures except two were graft failures at the mid-substance. Each group had one clavicular fracture just distal to the grip. These specimens were not included in the statistical analyses. Load to failure was significantly higher in group 1 compared to group 2 (Figure 4), with mean values of 437.5 N ± 160.7 N and 94.4 N ± 43.6 N, respectively (*p* = 0.001). The difference in displacement to failure (Figure 5), though higher in group 1 compared to group 2, with mean values of 29.7 mm ± 10.6 mm and 25 mm ± 9.1 mm, did not reach clinical significance (*p* = 0.25).

There was a 363% increase in average load to failure in UHMWPE augmented tendon grafts compared to the nonaugmented group. An increase in average displacement to failure was noted to be 19% in the augmented group.

## Discussion

The AC ligament, the CC ligaments (trapezoid and conoid), and to some degree the coracoacromial (CA) ligament, are the primary static stabilizers of the AC joints [2]. Therefore, high-energy injury to these structures will result in clinical instability and disability. Various operative techniques exist for management of AC joint separation. The goal is to maintain the AC joint reduction for an adequate period of time to allow healing [29]. However, recurrence rates of AC joint separation after reconstruction can range between 20–30% and higher [3, 14–18].

Recently the focus has been on repair using tendon grafts. In this study, we determined whether a difference in strength of AC joint repair exists between the use of a standard tendon graft and a tendon graft augmented throughout with UHMWPE suture.

As expected, our data supports the proposed novel technique to run UHMWPE suture throughout the entire length of the tendon graft for reinforcement. One explanation for why constructs using such augmented tendon grafts are stronger than nonaugmented is the added material that leads to greater time zero strength. Results of this study show that the proposed tendon graft augmentation technique significantly increases load to failure (356%).

Grutter and Petersen [30] compared various AC joint reconstructive techniques (modified Weaver-Dunn, anatomic reconstruction using palmaris longus tendon graft, and anatomic reconstruction using flexor carpi radialis tendon graft). One of their conclusions was that though the anatomical reconstruction is superior to the modified W-D reconstruction, the tendon graft used limited its strength. Load to failure using a flexor carpi radialis tendon graft was 774 N as opposed to 326 N using a palmaris longus tendon. The strength in the native AC joint (815 N) was greater than in any of their investigated reconstruction methods [30].

In this current study a substantial improvement in graft performance when augmented with a suture within the graft was observed. The maximum load to failure recorded in the augmented group was 438 N. The tremendous increase compared to the nonaugmented group (94 N) suggests that our method may be a viable option to improve the biomechanical strength of tendon grafts and provide longevity to AC joint repairs.

Charlick and Caborn [31] developed a graft preparation technique for cruciate ligament reconstruction usable in a wide range of grafts. The basis of their method is the circular placement of sutures (Whip stitch, Krackow stitch, or Baseball stitch) at the portion of the graft that will pass through a bone tunnel. This permits interdigitation of screw threads and the suture and subsequently provides protection of the graft from screw damage and increases pullout strength [31]. Our suggested method of placing sutures continuously through the entire length of the graft offers greater load to failure and may also lead to greater pull out strength when used with screws in anatomic AC joint reconstruction.

Concerns have been raised regarding clavicular fractures. Costic et al. [11] reported in their cadaver study two failed specimens due to clavicular fracture. One fracture occurred at the site where the clavicle was anchored in the epoxy compound. The second was due to inadequate bone bridge between the two bone tunnels in the clavicle. Turman et al. [32] reported on clavicular fractures after CC ligament reconstruction with a tendon graft. Suggested explanation of fractures includes placement of bioabsorbable screws that have the potential for osteolysis, insufficient patient compliance with the postoperative protocol, and imperfect communication between the surgeon and the patient regarding patient compliance with the postoperative protocol. Another factor seems to be the relatively large bone tunnels and subsequent cortical breach. Carofino and Mazzocca [2] noted that spacing the bony tunnels at least 20–25 mm apart could prevent clavicular fractures. If tendon grafts are reinforced with UHMWPE sutures, the graft diameter could be decreased without losing graft strength. Decreased graft and bone tunnel diameter will result in less clavicular substance loss and a larger bone bridge between the tunnels. This is something to be explored in future biomechanical studies.

In this biomechanical study, none of our samples fractured at the bone bridge between the tunnels. Each group experienced one clavicular fracture just distal to the part where the custom-made grips grabbed the clavicle. This may be due to the fact that our cadaver specimens had a higher average age (86 years) than the patients who generally require this procedure. To compensate for advanced age and possible osteoporosis the fractured specimens were excluded from statistical analysis.

Current graft augmentations, such as metal or suture cerclage techniques, have been linked to erosion of the coracoid and clavicle [33–35]. The incorporation of the suture into a tendon graft may potentially prevent the suture from cutting through clavicular tunnels or coracoid and thereby also prevent subsequent reoperations. Secondary surgeries for hardware removal, needed when employing coracoclavicular screw and plate techniques, are also obviated with our method [25, 35]. In addition, complications associated with breakage and migration of metal implants are avoided.

Our method may offer greater biologic fixation compared to all synthetic grafts and may also restore normal arthrokinematics because it is strong but of nonrigid nature, which is shown by the lack of statistically significant displacement to failure when compared to the nonaugmented tendon graft.

Almost half of all shoulder injuries in athletes involved in contact sports are AC joint injuries [36–38]. Also, surgical treatment of Type III is preferred in young active patients, manual workers, and high-level athletes [39]. This shows that higher energy injuries are more likely the cause of AC joint separations. Therefore, due to the limited number of specimens available, the decision was made to determine load to failure without number of cycles to failure. Even though the relatively stiff #5 FibreWire^TM^ has been added to the tendon graft, displacement to failure did not change significantly. This might be partially explained by the relatively circular shape of the stitch loops that changed to an oval shape with increasing tension. Cyclic loading may provide greater insight and should be the focus of future investigations.

Another limitation was the use of calf tendon for reconstruction, which may be slightly different from human-derived tendon grafts. Ideally, tendon grafts similar to those used in actual patient surgeries, for example semitendinosus allografts should have been used. However, since the basic microarchitecture of collagen fibrils is shared among mammals, there is no difference between the strength of individual bovine tendon fibers compared to human fibers. Therefore the grafts should be of similar strength, if the dimensions of the grafts are similar [40]. Also the focus of this study, being on a graft augmentation method and not on reconstruction techniques, allows the usage of bovine Achilles tendon.

## Conclusion

The results of our tendon graft augmentation method are very promising in terms of reconstructive strength in both maximum load and displacement to failure.

This method may be used with different AC joint reconstruction techniques that use tendon grafts for repair. However, further biomechanical and clinical studies using human allografts are warranted to explore the feasibility of our novel method.

## Conflict of interest

All authors certify that they have no financial conflict of interest (e.g., consultancies, stock ownership, equity interest, patent/licensing arrangements, etc.) in connection with this article. No benefits in any form have been received or will be received from a commercial party related directly or indirectly to the subject of this article.
